# Audit of VTE prophylaxis prescribing preferences among orthopaedic consultants in Irish orthopaedic trauma centres

**DOI:** 10.1007/s11845-025-03909-4

**Published:** 2025-02-21

**Authors:** Charles Timon, Conor Kilkenny, Nicola Byrne, John F. Quinlan, Niall P. McGoldrick

**Affiliations:** Tallaght University Hospital, Dublin, Ireland

**Keywords:** Ireland, Orthopaedics, Trauma, Venous thromboembolism, VTE

## Abstract

**Background:**

Thromboembolic complications are common in trauma orthopaedic practise. Despite extensive research, there remain a number of unanswered questions regarding the use of thromboprophylaxis.

**Aims:**

To establish the current practise among Irish consultant orthopaedic surgeons regarding thromboprophylaxis.

**Methods:**

A confidential online questionnaire regarding VTE prophylaxis was circulated to all consultant orthopaedic surgeons in training hospitals in the Republic of Ireland. Questions investigated surgeon awareness of local/national guidelines, inpatient and outpatient prescribing preferences and agreement/disagreement with the statement that defensive medicine, rather than evidence-based medicine, has driven increased emphasis on VTE prophylaxis in Ireland in recent years.

**Results:**

The response rate was 72% (69/96). 72% (50/79) of surgeons were aware of local VTE prophylaxis guidelines. 38% (29/96) were aware of national guidelines. 89% (62/69) routinely used mechanical prophylaxis modalities. 73.9% (51/69) routinely used chemical prophylaxis for inpatients. However, this practise was z extremely heterogenous with five other pharmacological agents used by the remainder. 82.6% (57/69) routinely discharged patients on extended duration chemical prophylaxis. 67% (46/69) agreed that the recent increased emphasis on VTE prophylaxis was due to defensive medicine and not evidence-based medicine.

**Conclusion:**

The results of this survey show that venous thromboembolism is regarded as a significant complication of orthopaedic surgery and most orthopaedic surgeons actively try to prevent it. There was a higher rate of intervention compared to previous surveys of Irish orthopaedic surgeons, possibly reflecting surgeon concerns regarding the high rate of litigation nationally. However, there is no consensus as to the optimum therapy which reflects the conflicting evidence available in the many publications on this subject.

## Introduction

Venous thromboembolism (VTE) is a common complication in trauma orthopaedic practise with rates of as high as 80% in polytrauma patients with femur fractures [1]. Other orthopaedic practises such as using cast immobilisation or restricting patients’ weight bearing status are associated with increased rates of VTE [2]. Furthermore, our patients are living longer and developing more comorbidities associated with increased risk of VTE [2]. In patients with less severe injuries, the prevalence is lower and the majority of these thrombi resolve without issue. However, a small percentage will develop into symptomatic VTE. Symptomatic VTE can result in symptoms ranging from mild ankle swelling to debilitating venous claudication or leg ulcers; they can propagate to develop pulmonary embolisms resulting in pulmonary hypertension, heart failure and even death [2]. Despite the many studies reported in the literature, there remains a number of unanswered questions regarding the use of thromboprophylaxis in orthopaedic trauma care including selection of the optimal agent and duration. The aim of this study was to establish the current practise among Irish consultant orthopaedic surgeons regarding thromboprophylaxis.

## Methods

A confidential online questionnaire regarding VTE prophylaxis for orthopaedic trauma was sent by email to all consultant orthopaedic surgeons in training hospitals carrying out orthopaedic trauma surgery in the Republic of Ireland. Google Forms was used to distribute and collect the data. Collection began on 01/06/2024 and ceased on 30/06/2024. Participants were asked the 13 following questions and also allocated a text box for further comments.

1. What hospital are you currently working in?
2. Are you aware of any written guidelines for VTE prophylaxis in your hospital?
3. Are you aware of any national guidelines for VTE prophylaxis post orthopaedic surgery for adult, trauma patients?
4. Do you routinely use/encourage the use of mechanical prophylaxis in adult, trauma inpatients?
5. If you answered Yes to Question 4, please specify what type of mechanical prophylaxis you use.
6. What agent do you primarily use for chemical VTE prophylaxis in adult, trauma inpatients?
7. When do you restart therapeutic anticoagulation (for example warfarin/direct oral anticoagulants) postoperatively?
8. Does your institution have protocols in place for the reversal of anticoagulation in adult, trauma patients who require surgery?
9. Do you routinely discharge adult, trauma patients at risk for VTE on extended duration chemical VTE prophylaxis?
10. If you answered Yes to Question 9, what patient populations do you discharge on extended duration chemical VTE prophylaxis? (Tick all that apply)
11. If you answered Yes to Question 9, what agent do you routinely use for extended duration chemical VTE prophylaxis in the outpatient setting post hospital discharge?
12. If you answered Yes to Question 9, how long do you continue extended duration chemical VTE prophylaxis in patients at risk of VTE in the outpatient setting?
13. To what extent do you agree or disagree with the following statement: Defensive medicine, as opposed to evidence-based medicine, has led to an increased emphasis on VTE prophylaxis in Irish trauma orthopaedics in recent years.

Ethical review and approval were not required for the study in accordance with the local institutional requirements.

## Results

The response rate was 72% (69/96). Surgeons from 16 different training hospitals in Ireland responded.

### Guidelines

**Fig. 1:**
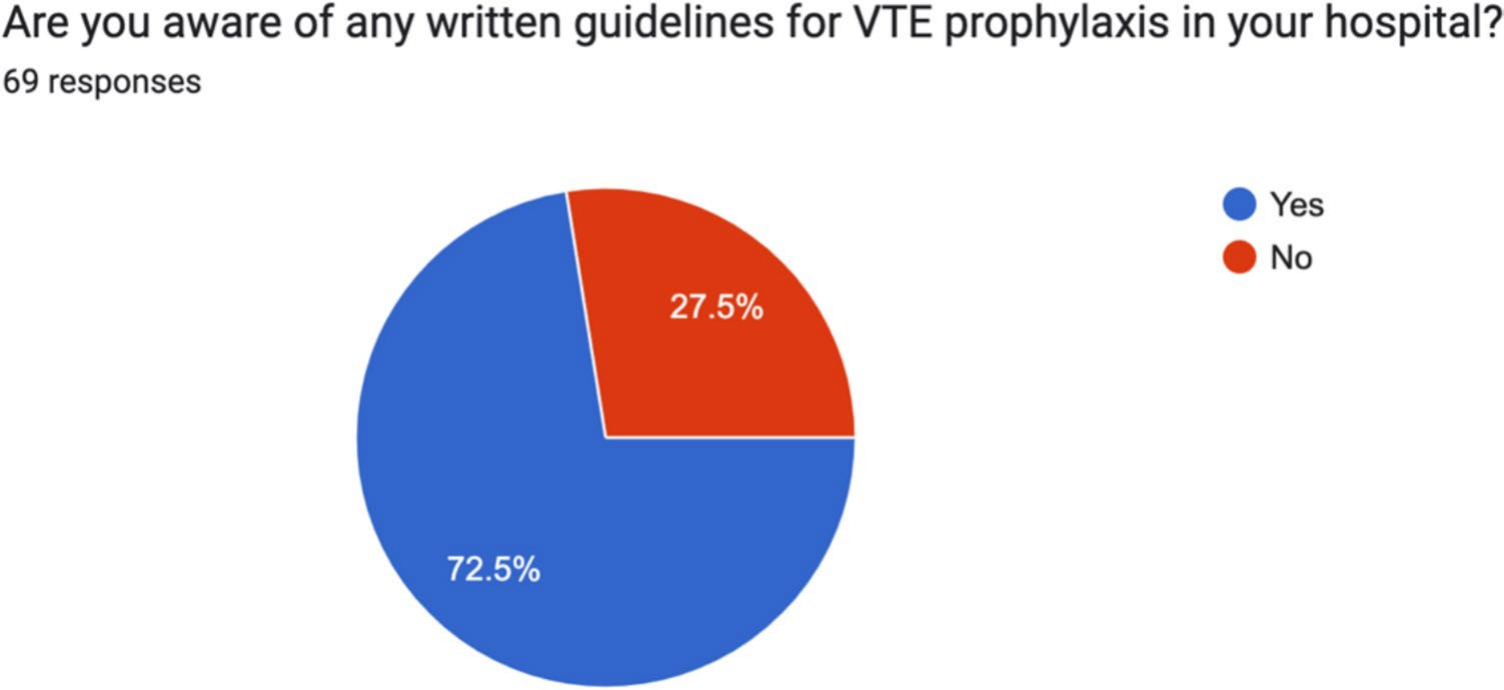
Irish consultant orthopaedic surgeon awareness of written guidelines for VTE prophylaxis in their hospital

**Fig. 2:**
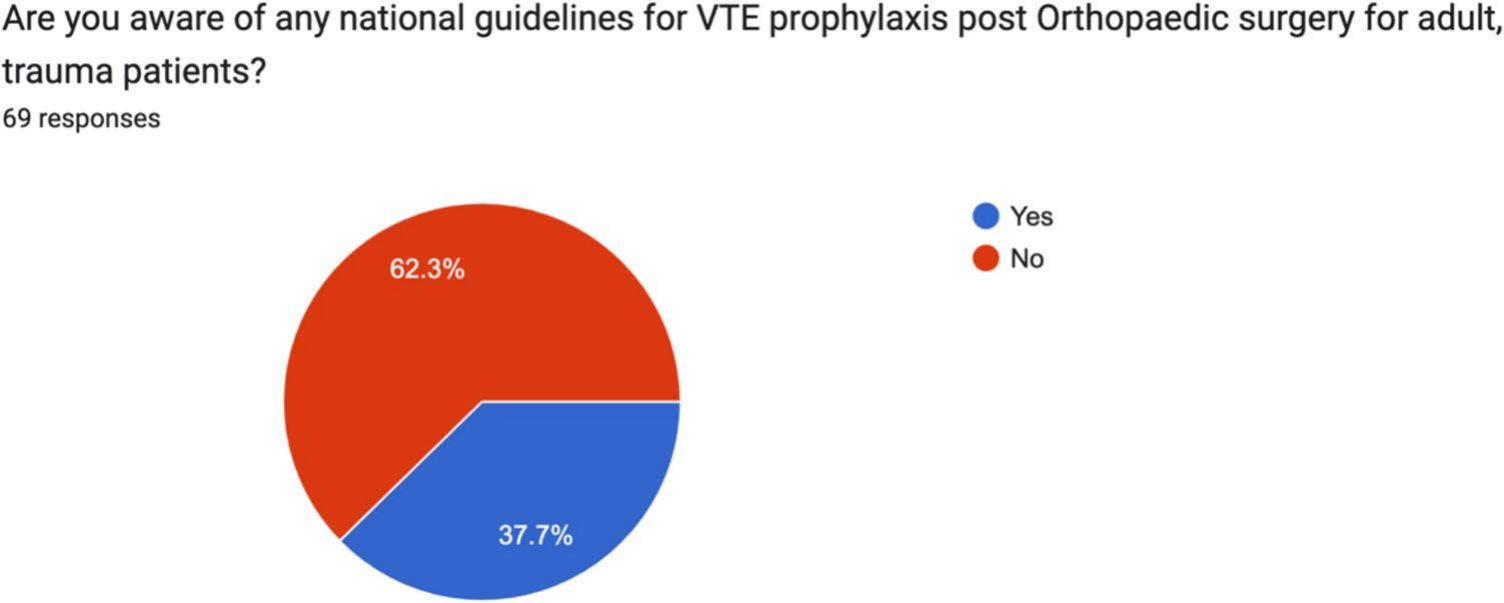
Irish consultant orthopaedic surgeon awareness of national written guidelines for VTE prophylaxis for trauma patients

72.5% (50) answered that they were aware of written guidelines for VTE prophylaxis in their hospital. 37.8% (26/69) answered that they were aware of national guidelines for VTE prophylaxis post orthopaedic surgery for adult trauma patients. At the time of data collection, no national guidelines were available in Ireland (Figs. 1 and 2).

### Inpatient prescribing preferences

**Fig. 3:**
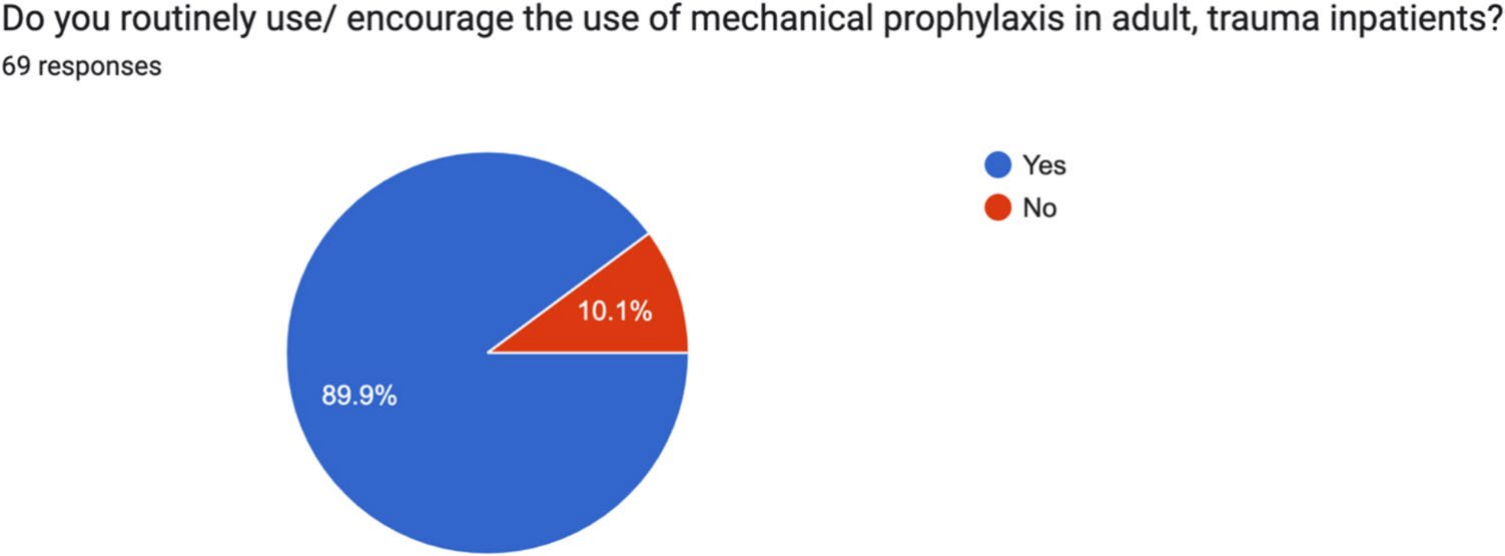
Irish consultant orthopaedic surgeon use of mechanical prophylaxis in adult, trauma inpatients

**Fig. 4:**
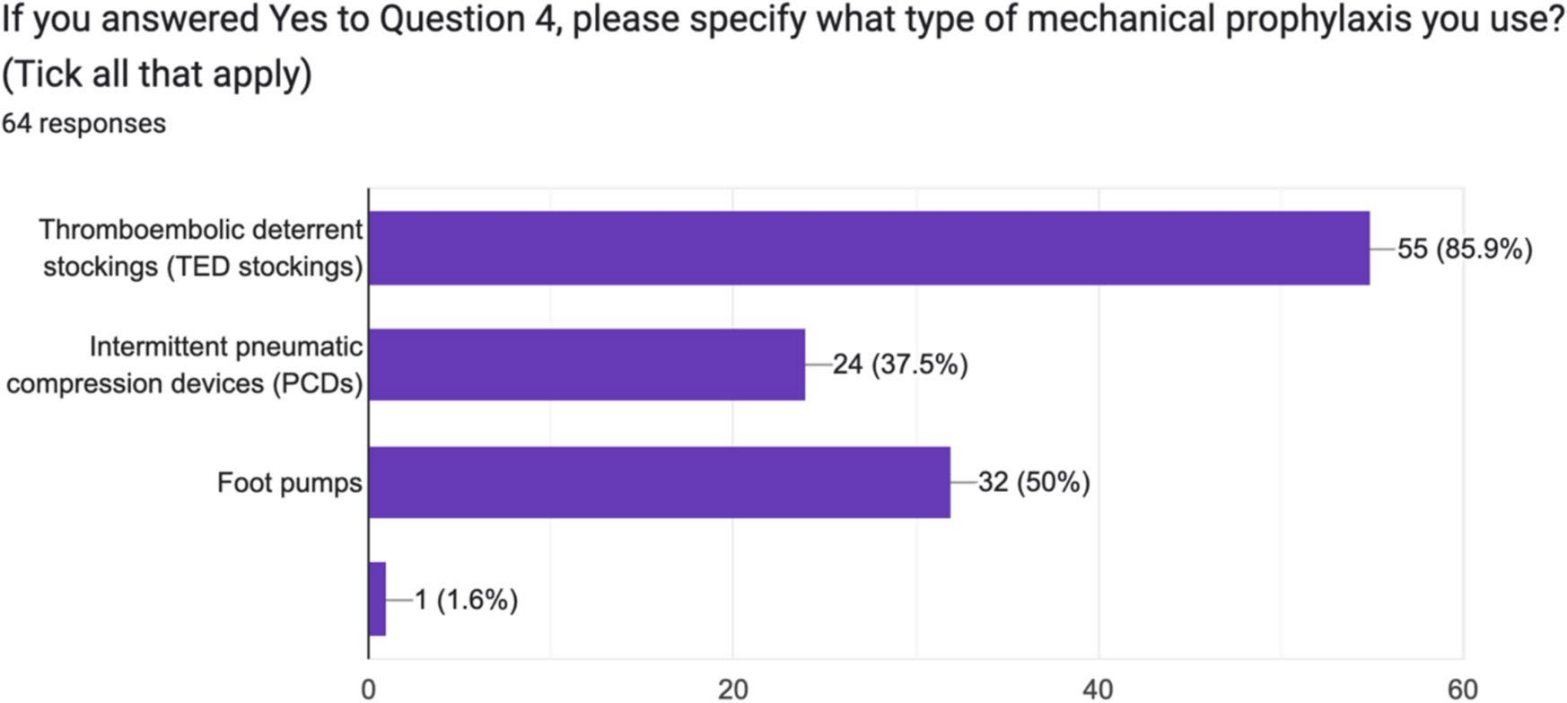
Breakdown of device used by Irish consultant orthopaedic surgeon for mechanical prophylaxis in trauma inpatients

**Fig. 5:**
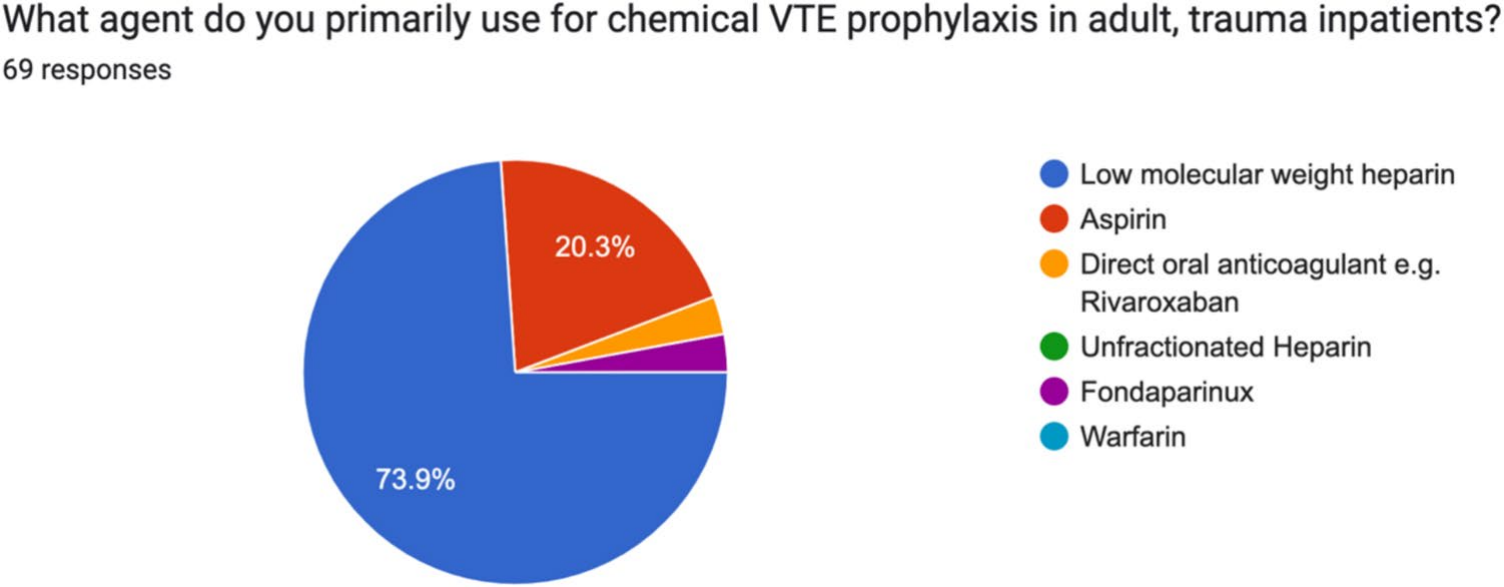
Breakdown of agent used by Irish consultant orthopaedic surgeon for chemical VTE prophylaxis

90% (62) of surgeons routinely used mechanical prophylaxis in adult, trauma inpatients. On further analysis, 85.9% (55/69) routinely used thromboembolic deterrent stockings (TEDs), 37.5% (24/69) routinely used intermittent pneumatic compression devices (PCDs) and 50% (32/69) routinely used foot pumps. 100% of respondents used chemical VTE prophylaxis in adult, trauma inpatients. 74% (51/69) used low molecular weight heparin (LMWH), and 20.3% (14/69) used Aspirin. Two surgeons each (3%) routinely used a direct oral anticoagulant (DOAC) and one routinely used Fondaparinux (Figs. 3, 4 and 5).

**Fig. 6:**
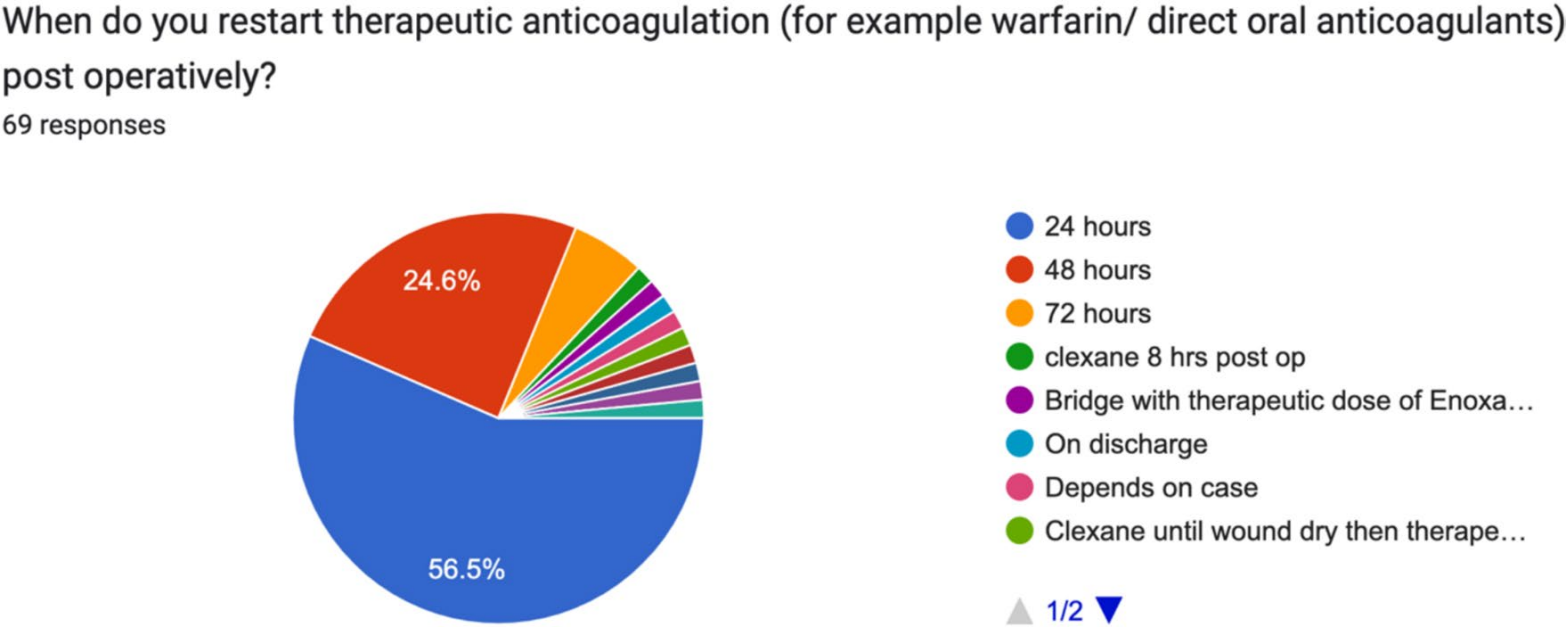
Timing of restarting therapeutic anticoagulation postoperatively by Irish consultant orthopaedic surgeons

When asked about restarting therapeutic anticoagulation, e.g. warfarin or a DOAC postoperatively, 56.6% (39/69) restarted it 24 h postoperatively, 24.6% (17/69) restarted it at 48 h and 7% (4/69) of surgeons restarted therapeutic anticoagulation at 72 h. A comments option for this question showed further practises and comments included ‘depends on case’ and also different timelines ‘if wound is dry’ (Fig. 6).

**Fig. 7:**
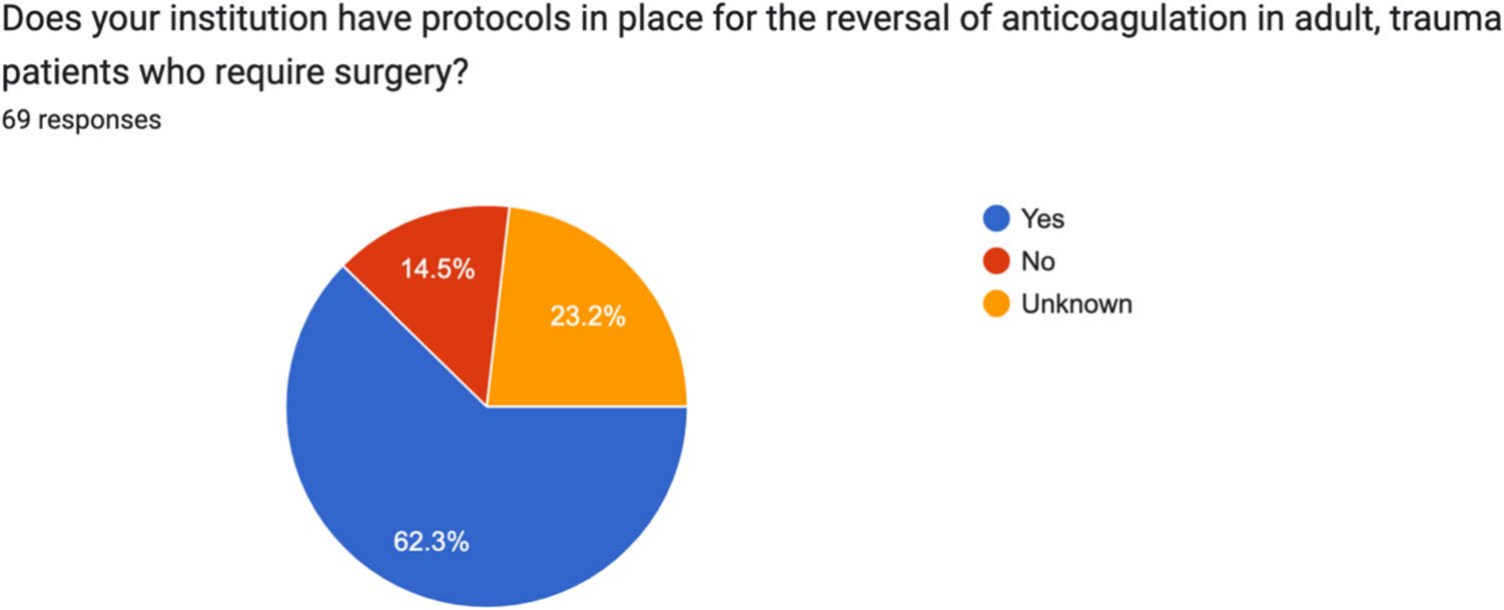
Irish consultant orthopaedic surgeon awareness of written guidelines for reversal of anticoagulation for adult trauma patients requiring surgery

When asked whether their respective hospitals had protocols in place for the reversal of anticoagulation in adult, trauma patients who required surgery, 62.3% (43/69) responded yes, 14.5% (10/69) responded no and 23.2% (16/69) did not know (Fig. 7).

### Outpatient prescribing preferences

**Fig. 8:**
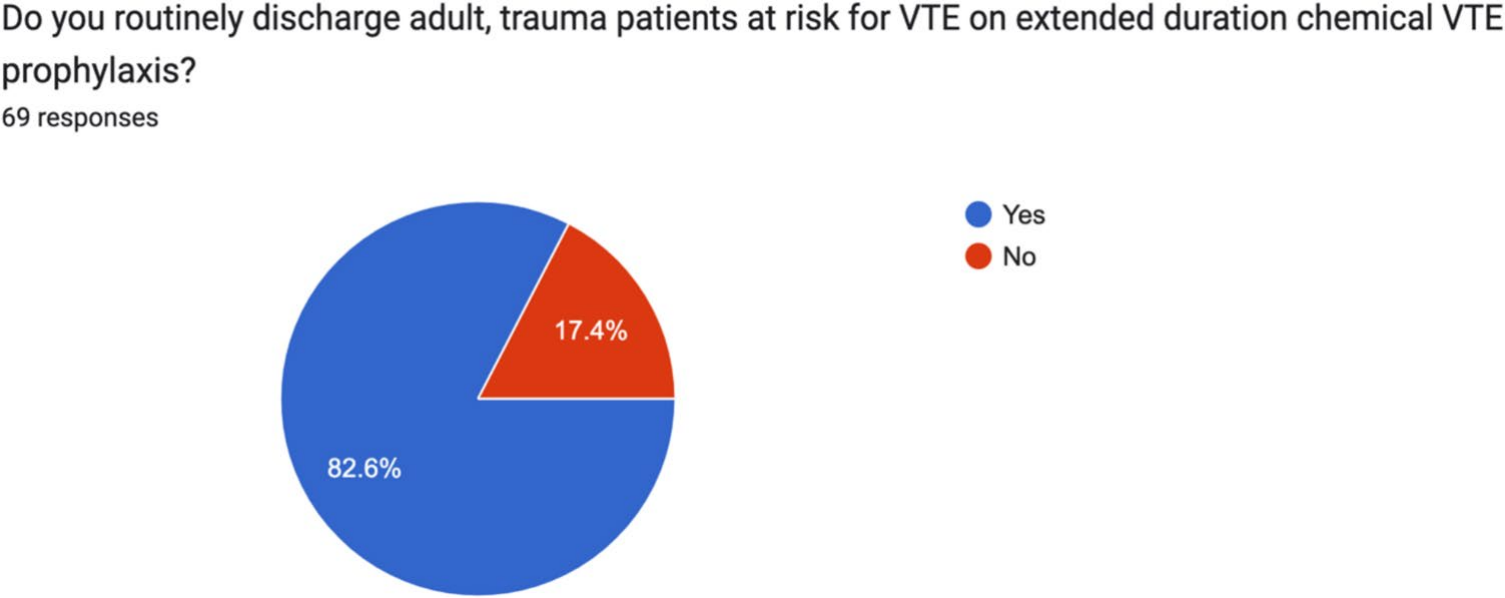
Breakdown of practise by Irish consultant orthopaedic surgeons with regard to discharging patients on extended duration chemical VTE prophylaxis

**Fig. 9:**
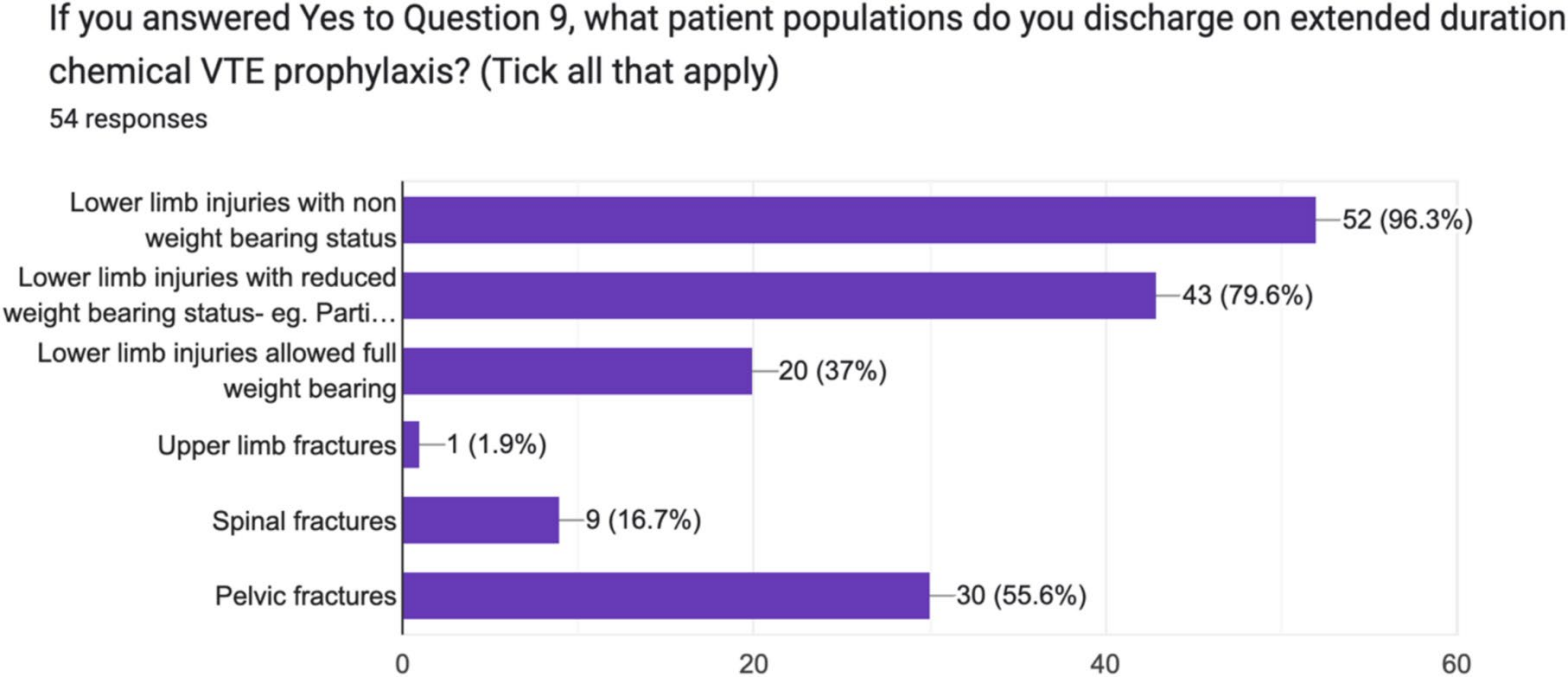
Prescribing preferences regarding patient selection for extended duration chemical VTE prophylaxis by Irish consultant orthopaedic surgeons for trauma patients

82.6% (51/69) surgeons routinely discharged adult, trauma patients at risk for VTE on extended duration chemical VTE prophylaxis. This question was further analysed, however, with varying response rates, and for non-weight bearing lower limb injuries, the proportion was 96.3% (52/54), 79.6% (43/54) for lower limb injuries prescribed reduced weight bearing and 37% (20/54) for lower limb injuries allowed fully weight bear. 16.7% (9/54) of consultants discharged spinal fractures on chemical VTE prophylaxis and 55.6% (30/54) discharged pelvic fractures on chemical VTE prophylaxis (Figs. 8 and 9).

**Fig. 10:**
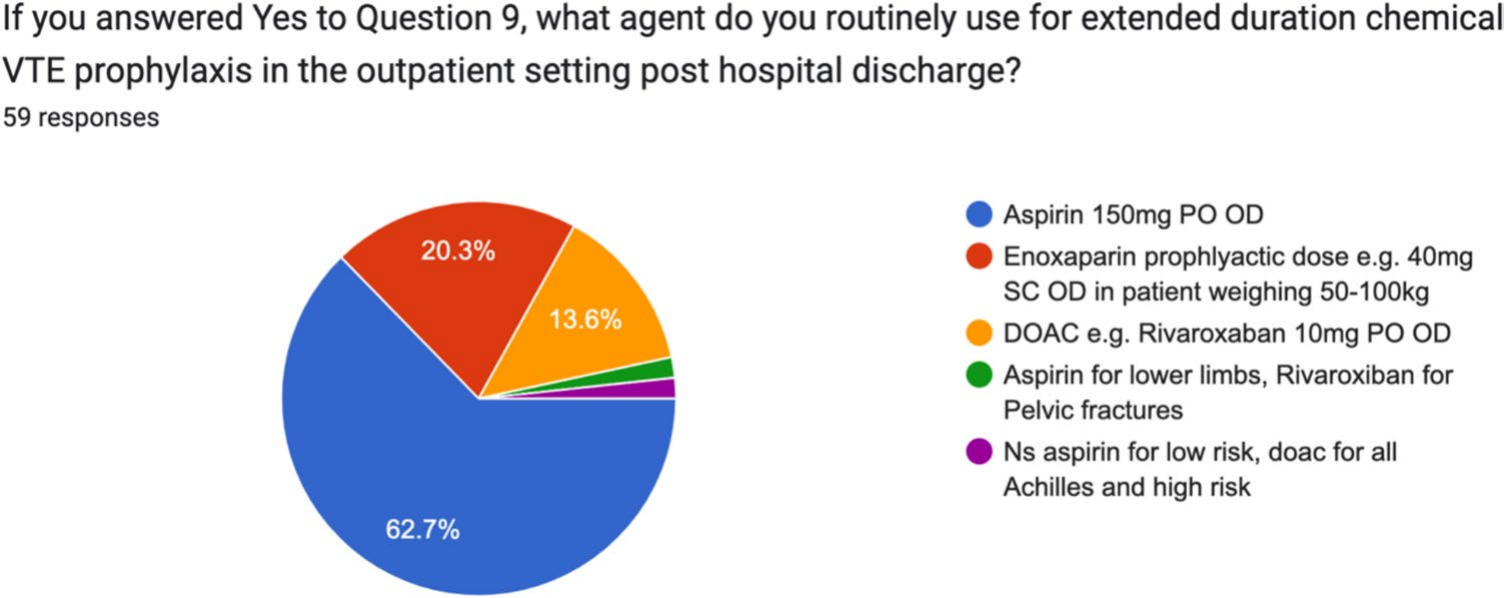
Prescribing preferences regarding agent of choice for extended duration chemical VTE prophylaxis by Irish consultant orthopaedic surgeons for trauma patients

**Fig. 11:**
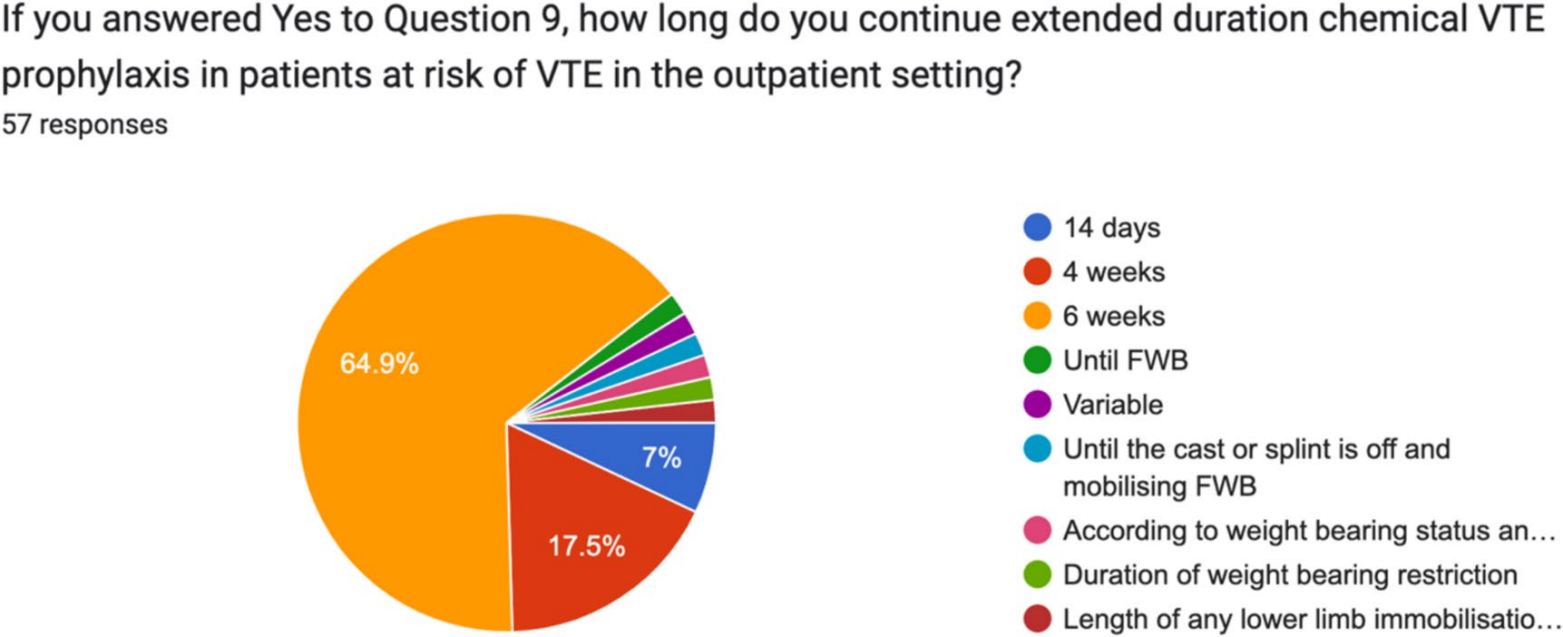
Prescribing preferences regarding duration of extended duration chemical VTE prophylaxis by Irish consultant orthopaedic surgeons for trauma patients

For extended duration chemical VTE prophylaxis, 65.9% (37/59) of surgeons used Aspirin 150 mg PO OD, 20% (12/59) surgeons used prophylactic dose Enoxaparin (40 mg SC OD in patients weighing 50–100 kg) and 13.6% (8/59) of surgeons used a DOAC (Figs. 10 and 11).

### Current attitudes

**Fig. 12:**
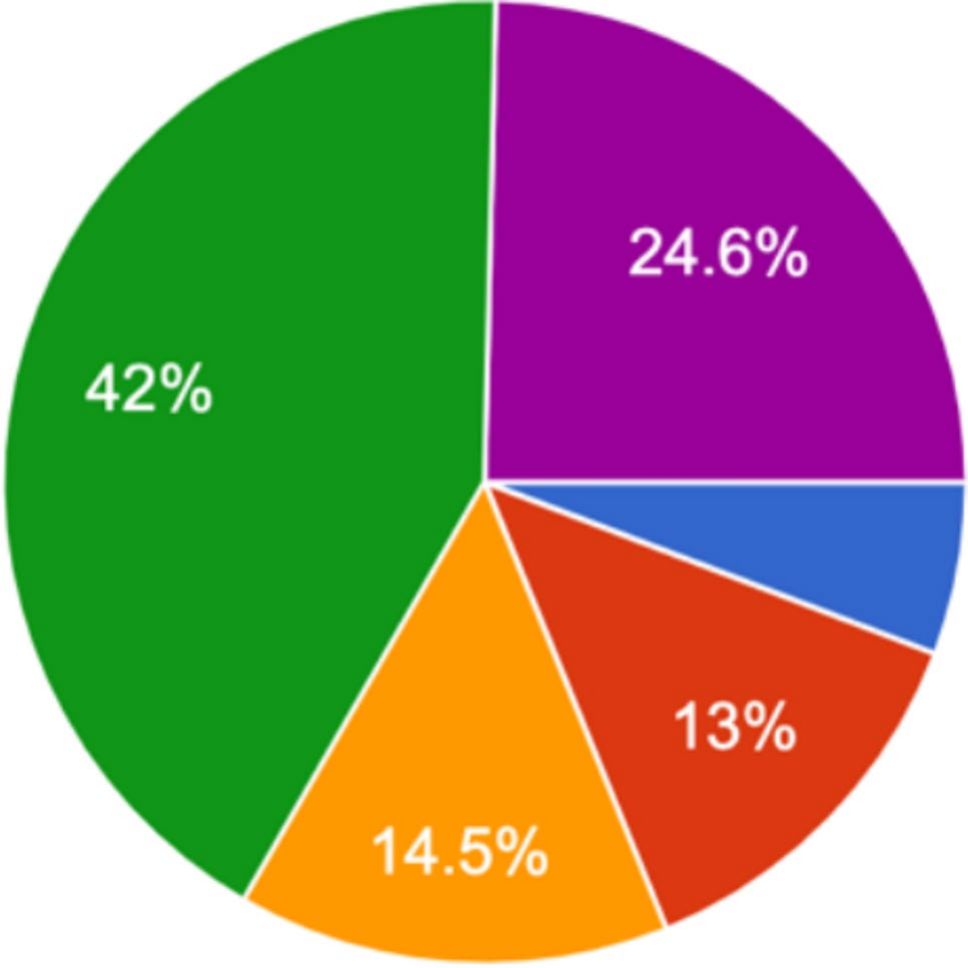
Graphical representation of agreement/disagreement by Irish consultant orthopaedic surgeons with the statement ‘Defensive medicine, as opposed to evidence-based medicine, has led to an increased emphasis on VTE prophylaxis in Irish trauma orthopaedics in recent years.’

When asked as per the 5-point Likert scale [3] to what extent they agreed/disagreed with the statement ‘Defensive medicine, as opposed to evidence-based medicine, has led to an increased emphasis on VTE prophylaxis in Irish trauma orthopaedics in recent years.’, 24.6% (17/69) strongly agreed, 42% (29/69) agreed, 14.5% (10/69) were neutral, 13% (9/69) disagreed and 5.8% (4/69) strongly disagreed (Fig. 12).

An option for further comments was attached to the survey and is addressed in the discussion.

## Discussion

VTE post both operatively and non-operatively managed orthopaedic injuries results in significant morbidity and mortality [4]. The prevalence of deep vein thrombosis (DVT) and pulmonary embolism (PE) in orthopaedic patients is estimated at 1.16% and 0.93% respectively [5]. While the absolute risk is low, it manifests a considerable burden of disease when taking into account the annual caseload of Irish trauma surgeons; last available figures reported over 24,000 orthopaedic trauma admissions in 1 year and predicted this number as well as the average length of stay to increase due to our increasingly ageing population [6]. The results of this survey show that most orthopaedic surgeons take active steps to try and prevent its occurrence. Pharmacological prophylaxis may reduce the risk of VTE by up to 50%. However, there is a lack of evidence that it reduces the risk of fatal PE in orthopaedic patients [7]. Unfortunately, however, there have been a small amount of high-profile cases in Ireland, in which patients suffered significant morbidity or passed away due to VTE while not on prophylactic treatment. Despite the lack of evidence for these treatments, their treating orthopaedic doctors were subject to disciplinary and litigation procedures.

The Irish health service (Health Service Executive) has a guideline for VTE prophylaxis in patients post total hip arthroplasty and total knee arthroplasty [8], but not for orthopaedic trauma patients. Many Irish orthopaedic surgeons refer to the UK’s NICE guidelines [9, 10]. These guidelines and other countries’ guidelines may explain the heterogeneity in question 3 above. The lack of national robust guidelines leaves surgeons alone to interpret conflicting evidence and, as can be seen above, has resulted in a massive heterogeneity of practise. VTE following upper limb trauma is much reduced compared to lower limb trauma (Hsu, 2011 #437). This may also contribute to the massive heterogeneity in practise. There was a higher rate of intervention in this group of surgeons compared to previous surveys of Irish orthopaedic surgeons [11]. This may reflect surgeon concerns regarding the high rate of litigation in the Republic of Ireland. The economic impact of VTE is enormous: it is estimated that VTE is associated with annual costs of €640 million per year in the UK National Health Service, but data from the state claims agency is not currently available [12].

Orthopaedic surgeons are required to make a decision balancing the individual patient’s risk of VTE and bleeding. Risk assessment models have been designed to support this but have not been validated [13]. In the UK, cost-effective analyses have shown that broad prescribing for all eligible patients (i.e. patients without contraindications) is cost-effective [14].

The next issue to address is what to prescribe. UK guidelines recommend ‘considering parenteral pharmacological prophylaxis’, with ‘considering’ the key term as there are significant limitations in available evidence and subcutaneous administration of LMWH is inherently unpleasant. Recent landmark trials include the PRONOMOS study and the PREVENT-CLOT study. The PRONOMOS study showed that Rivaroxaban is superior to low molecular weight heparin (LWMH) in patients undergoing postoperative immobilisation. PRONOMOS study also demonstrated a similar risk profile for bleeding, and these findings combined with its ease of administration are appealing; however, there are concerns regarding its inclusion criteria which could affect its generalisability for individual patients [15]. PREVENT-CLOT concluded that Aspirin is non-inferior to LMWH with regard to all-cause mortality. However, the absolute risk of DVT with Aspirin actually increased; this was also seen in the CRISTAL trial, a noninferiority trial comparing Aspirin to LWMH in arthroplasty patients in Australia [16].

It is difficult to know where to proceed from here. Until this guidance is clarified, orthopaedic surgeons in Ireland should work within recommendations from their hospitals to allow consistent, but most importantly defensible practises.

## Conclusion

The results of this survey show that venous thromboembolism is regarded as a significant complication of orthopaedic surgery and that most Irish orthopaedic surgeons take active steps to try and prevent its occurrence. However, there is a massive heterogeneity in practise which reflects the conflicting evidence available in the literature on this subject. To protect both patients and orthopaedic surgeons, robust national guidelines based on the available evidence are needed.
